# Nurse lecturers’ perceptions of their professional dignity: A phenomenological study

**DOI:** 10.1177/09697330251366597

**Published:** 2025-08-14

**Authors:** Christelle Froneman, Neltjie van Wyk, Varshika Bhana-Pema

**Affiliations:** Department of Nursing Science, 56410 University of Pretoria, Pretoria, South Africa

**Keywords:** Educational management, phenomenology design, professional dignity, empowering environments

## Abstract

**Background:**

Lecturers who are working in empowering environments and are treated with respect by students and managers, feel dignified and capable of being positive role models to their students. The opposite happens when they are not treated with respect and experience challenging working environments.

**Aim:**

The aim of the study was to explore and describe how nursing lecturers of a designated education institution in South Africa experienced factors that influenced their perceived professional dignity.

**Research design:**

A descriptive phenomenological research design with a constructivist paradigm applied. Individual interviews were conducted with lecturers to obtain answers to the question *‘How do you experience factors at the institution that influence your dignity as a professional person?’* Probes were used encouraging the participants to comprehensively describe their experiences. The data were analysed according to the steps designed by Giorgi in which the natural dimension of the phenomenon as narrated by the participants was transformed into the phenomenological dimension of the phenomenon.

**Participants and research context:**

The study was undertaken in South Africa at a nursing education institution and 18 voluntary purposively selected lecturers were involved.

**Ethical considerations:**

The Faculty of Health Sciences Research Ethics Committee at the University of Pretoria approved the proposal and the applicable authorities gave written permission for the research to be conducted. Informed voluntary consent was obtained from the participants before the research commenced.

**Findings:**

The essence of the participants’ experiences refer to ‘longing for professional dignity.’ The supporting constituents are: ‘acknowledging lecturers as specialists’, ‘enhancing their self-determination’, ‘acknowledging their capabilities’, ‘promoting collegiality’, ‘creating conducive teaching environments’, ‘being respected by students’, and ‘perceiving lecturers as possessing integrity’.

**Conclusion:**

The participants’ perceived dignity was influenced by the way they had been treated by students and managers at the designated institution. Their working environment also impacted on their perceived professional dignity.

## Introduction and background

Dignity is deeply imbedded within many countries’ laws and constitutions.^1^ Although dignity is inherent, a distinction is made concerning the influences of the working environment and the interaction with people on the perceived professional dignity of nursing lecturers. The way they view themselves and the manner in which students and institutional managers interact with them may determine their perceived professional dignity.^2–6^

Respect for one’s dignity is as important as respect for others’ dignity, understanding what dignity means to a person is an important starting point.^7^ This pertains to values within an ethical framework.^8^ Nursing lecturers’ professional dignity is determined by their professional image (how they portray themselves and are perceived by others) and their professional identity (their beliefs, values and motivations).^2–5,8^ Dignified lecturers are needed to teach students how to uphold patient dignity and maintain quality patientcare.^9^

The perceived professional dignity of nursing educators should be affirmed by others in the working environment.^4,10^ When they are treated with respect, their perceived professional dignity could potentially be honoured and enhanced.^10–12^ An empowering working environment may positively influence the professional dignity of nursing lecturers.^10,11^ A positive sense of perceived professional dignity is associated with optimal functioning and quality of work.^9^ Teamwork and involvement in decision making processes improve the self-respect of individuals. Nursing lecturers who feel supported and respected by management,^11^ their colleagues and students, and benefit from an empowering working environment may enjoy an enhanced experience of professional dignity.^13^

## Problem statement

The perceived dignity of nursing lecturers may be violated when working in stressful situations.^14^ Examples are disrespect from students and not feeling supported by institutional management.^2^ Under such circumstances, they may find it difficult rendering quality training to students.^15^ Without respect and support from the Nursing Education Institution management, the lecturers may struggle to uphold their perceived dignity.^2,10,11,16^ High student numbers and negative student attitudes contribute to nursing lecturers’ challenging work environment.^17^ The researcher, a lecturer at the designated institution, noticed that she and her colleagues often had to cope with situations of unfavourable working environments.

## Aim of the study

This study aimed to explore and describe how nursing lecturers of a designated education institution experienced factors that influenced their perceived professional dignity.

### Research design

A descriptive phenomenological research design within a constructivist paradigm was used. The findings were co-created by the researcher and participants during individual interviews to explore and describe the participants’ experiences regarding their perceived dignity. The researcher was interested in the meaning that the participants attached to their experiences and not in a description of their experiences. After voluntary informed consent by the participants, the interviews took place in a private venue and at times convenient to them. One question was asked of all participants namely *‘How do you experience factors at the institution that influence (it may be positive or negative) your dignity as a professional person?’* Probes were used to encourage the participants to comprehensively describe their experiences.

The data was analysed according to the recommendations of Giorgi.^18,19^ The researcher (first author) transcribed the interviews verbatim and indicated the non-verbal gestures where applicable. The data was then read and slowly re-read by the researcher to familiarize herself with the content. Through a process of ‘bridling’ premature understanding of the transcripts was prevented. Once the essence of the studied phenomenon was revealed, the constituents (also called meaning units) that substantiate the essence were identified. The data analysis program ATLAS. ti version 23 was used to assist with verifying the meaning units.^20^ ATLAS. ti assists with working through large amounts of information that can be linked to constituents.^20,21^ Verbatim quotations from the interviews were used to corroborate the data with the constituents.

### Participants and research context

The study was conducted in a designated nursing education institution in the Gauteng Province of South Africa. At the time of the research, there were 82 lecturers, with ages from 29 to 63 years, all qualified in nursing education with 2 years of experience. A purposive sampling method using maximum variation to capture the widest possible range of perspectives and experiences related to the research question, was chosen based on the premise that the population has varying levels of experience of the phenomenon and ranges in age^18,22,23^ 18 participants were interviewed until data saturation was reached.

## Demographic information of the participants

The ages of the 18 participants ranged between 31 to 35 and 61 to 65 years. Only one male participant was interviewed. In South Africa the majority of nursing lecturers are female. The participants had vast experiences in nursing education. One participant had 36 years of teaching experience (refer to Table 1).

**Table 1.** Participants’ demographic information.

Participant	Age group	Gender	Years of teaching
Participant 1	51–55	Female	20–25
Participant 2	56–60	Female	35–40
Participant 3	51–55	Female	2–3
Participant 4	51–55	Female	6–10
Participant 5	51–55	Female	6–10
Participant 6	56–60	Female	6–10
Participant 7	56–60	Female	11–15
Participant 8	41–44	Female	6–10
Participant 9	51–55	Male	6–10
Participant 10	36–40	Female	2–5
Participant 11	36–40	Female	2–5
Participant 12	41–45	Female	2–5
Participant 13	61–65	Female	11–15
Participant 14	31–35	Female	2–5
Participant 15	61–64	Female	30–35
Participant 16	41–45	Female	2–5
Participant 17	56–60	Female	16–20
Participant 18	41–45	Female	6–10

*Note*: P = Participant number.

## Ethical considerations

Approval of the research was obtained from the Faculty of Health Sciences Research Ethics Committee of the University of Pretoria. The management of the applicable authorities gave permission for the research to be done at the designated nursing education institution, and the participants gave voluntary informed consent to be involved in the research. They were provided with a detailed explanation of the purpose of the research and were assured that they had the right to withdraw from the research at any time and that no information would be included in the research report by which they could be identified. The researcher used pseudonyms during the transcription of data so that no information could be linked to specific participants. The first author was at the time of the research a lecturer at the institution and was not in a supervisory relationship with any of the participants. She was open about her position in the institution and the participants did not object to share with her their experiences of factors that could influence their professional dignity as lecturers.

## Trustworthiness of the findings

Rigour in descriptive phenomenological research is ensured when researchers ‘bracket’ their pre-knowledge regarding the understanding of the studied phenomenon.^24,25^ The researcher applied bracketing of her perspectives of factors that may influence the professional dignity of lecturers to ensure that the phenomenon is studied in its pure form (refer to Table 2). Using imaginative variation during data analysis, phenomenological eidetic reduction with the use of bracketing was applied to convert all the data gathered from the participants into the central essence. It brought out the meaning of the phenomenon (the perceived dignity of the nursing lecturers and the factors that influenced it).^21,26^

**Table 2.** Bracketing with the use of reflective notes/journal.

The researcher forms part of the population from which participants were selected for this research. I Acknowledge that as being part of this community of nursing lecturers, I know and see things that happen within the nursing education institution and thus, as I had previously done research on dignity and seen how dignity does not only include patient dignity but also the dignity of the professional nurses it was decided to replicate this phenomenological research within nursing education
• I Know from experience that autonomy given can promote your self-image and self-esteem
• I Experience factors that influence my dignity, either in positive or negative ways
• After and during the worst of the COVID-19 pandemic, the country’s president had thanked the nurses and everyone involved for training of nurses to assist with the large number of patients. This seemed like a very nice way of thanking, and I personally thought this would let lecturers feel dignified, but unfortunately, as I was conducting interviews, this was not what I found. Their perceived dignity had actually diminished as their working environment was placing stress on everyone within the institution
• What I personally believe and have seen to a certain extent is that when professional nurses are not respected, they are mostly unable to interact with others (patients and students) in a dignified manner
• Closer attention needs to be given based on the dignity of lecturers and training of excellent quality nurses and not just high quantity
• Bracketing was used successfully to ensure that during the interviews no bias comes in at that stage of the research

## Findings

The research revealed that several factors according to the participants influenced their perceived professional dignity. The essence of their experiences referred to ‘longing for professional dignity’. The meaning units, also called constituents in phenomenological research that support the essence of the experiences are: acknowledging nursing lecturers as specialists, enhancing their self-determination, acknowledging their capabilities, promoting collegiality, creating conducive teaching environments, being respected by students, and being perceived as possessing integrity (Refer to Table 3). The nurturing of their professional dignity existentially coincided with ‘being-for-self’ (or collectively as nursing lecturers ‘being-for-themselves’) and the factors that influenced their professional dignity coincided with ‘being-for-others’. They were also responsible for others, namely, the professional growth of students which is a facet of ‘being-for-others’.^27^ There was no data collected that the researcher could not link to the essence and its substantiating constituents.

**Table 3.** The essence and supporting constituents of factors that influence the perceived professional dignity of lecturers.

The essence: ‘Longing for professional dignity’	Constituents that were discovered that support the essence
	Perceiving nursing lecturers as specialists
	Enhancing self-determination
	Acknowledging the capabilities of lecturers
	Promoting collegiality
	Creating conducive teaching environments
	Being respected by students
	Perceiving lecturers as possessing integrity

The essence or the ‘new whole’ was revealed to the researcher within the phenomenological dimension and is substantiated by the constituents (meaning units).^24^ It provides new meaning and understanding of how nursing lecturers experience factors that influence their perceived dignity. The essence is described first, followed by the description of the supporting constituents.

## Essence

Nursing lecturers’ ‘being-for-themselves’ refers to their longing for professional dignity. It coincides with ‘being-for-others’ as they need to teach and guide students to provide dignified care to patients. Students being admitted into nursing programmes focus mostly on themselves, developing their chosen profession. Lecturers have the role of teaching, forming and moulding the future generation of professional nurses. The participants were conscious of their professional dignity being based on how others viewed them and how others’ perspectives influenced their own.

## Constituent: perceiving nursing lecturers as specialists

Nursing lecturers’ professional image as specialists in education contributes significantly to how they perceive their professional dignity. The participants’ professional image and thus their perceived dignity was according to them dependent on the acknowledgement of their qualification in nursing education as a specialist qualification. The participants felt that their nursing education qualification was not appreciated by others:

“I take my professional image as something very important…perceive dignity as having a professional image…” (P12)

“I think upholding the ethical prescripts of our profession also play a part in my dignity being upheld.” (P3)

While the lecturers’ professional dignity was not acknowledged, they had to ensure that the dignity of nursing students is upheld so that they can support patients’ dignity:

“As educators in teaching our students… for them not to forget the dignity of the recipient of our care.” (P3)

Nursing education is often considered as a non-specialist qualification while nurses who specialized in clinical areas such as trauma and acute care nursing are considered as true specialists. Nursing lecturers are therefore often not held in high regard:

“Master’s and PhD degrees are not even recognised by the South African Nursing Council and up till now has not had any effect on our salary scales… while others get paid extra for speciality qualifications in trauma and acute care nursing.” (P8).

## Constituent: enhancing self-determination

Self-determination focuses on personal growth and development. It enables individuals to make decisions on their own and to take responsibility for their actions. Self-determination is closely related to being able to function independently.

The participants wanted to be allowed to provide input into decisions made in the working environment. They wanted to take part in decision-making in the designated nursing education institution. That would have honoured their perceived professional dignity:

“To be given an opportunity to take part in the core function of the nursing education institution, which normally happens when lecturers are invited to managerial meetings and also when they take part in operational planning.” (P1)

The participants’ need for self-determination had not been respected as they never have had the opportunity to take part in decision making in the institution. They were expected to implement decisions made by others. The participants were convinced that their professional dignity could have been enhanced, should they had been given opportunities to take part in managerial processes:

“To give input in any planning that the management wanted to implement… when they show gratitude on your input and … implement the input, you have been shown respect that could have enhanced one’s professional dignity.” (P1)

“Then that opportunity to say something, to have a word, to have a say in the whole management of student learning.” (P15)

“Dignity means being given the opportunity to practice independently, to make decisions independently… It will give me the… confidence to continue practising what I do best.” (P12)

“Respect, treating every individual as a unique being… with their own ideas and how they … acknowledge our strengths and our weaknesses and learn to improve.” (P3)

When nursing lecturers’ need for self-determination is respected and they be given opportunities to take part in decision making by the management of the nursing education institution, their sense of professional dignity gets enhanced. Nursing lecturers who are honoured for their decisions to specialise in nursing education, may therefore make huge contributions to the training of future professional nurses to the benefit of the profession.

## Constituent: to acknowledge the capabilities of lecturers

Nursing lecturers’ capabilities are used to enhance the development of their students. Their capabilities include personal, communication and technical skills as well as their nursing knowledge and clinical skills.

In South Africa, the salary scale of nursing lecturers is lower than that of professional nurses with the same professional qualifications working in a hospital or clinic. Unfortunately, this caused many nursing lecturers to change their careers as they could earn higher salaries in clinical posts:

“In general, speciality posts at hospitals and clinics pay better than at nursing education institutions and I think in terms of professional dignity as professional persons, nursing lecturers’ salaries need to be improved.” (P2)

Acknowledgement of lecturers’ capabilities may lead to upholding and honouring their perceived professional dignity. It may also promote lecturers’ job satisfaction and decrease the education institutions’ staff turnover rate. The participants felt that the acknowledgement of their capabilities played a vital part in enhancing their perceived professional dignity:

“Dignity to me means receiving respect, being acknowledge and being considered as a valuable employee, a subordinate and a person whose giving input to and enriching the institution.” (P1)

The participants felt that the institution’s management often underestimated their competencies and the contribution that they made to benefit the institution and its students. They wished that their capabilities in teaching students and co-managing the institution could be acknowledged.

## Constituent: promoting collegiality

Collegiality is often described as working in supportive environments and having good relationships with colleagues. In nursing education, collegiality has a deeper connection than just the working relationship between colleagues. In order to teach, develop, mentor and mould future generations of ethically sound and caring professional nurses, it is essential that lecturers display similar behaviour and, therefore, cooperate with and respect their colleagues:

“As colleagues we really respect each other… we really have good relationships and teamwork.” (P6)

Management can increase the effectiveness of interaction among team members in order to create mutual trust, improve interpersonal relationships and enhance employee job satisfaction. One participant described it as follows:

“Part of dignity and being respected is to be heard, to be allowed to raise concerns and feel that you are being listened to and you are having a say in day-to-day activities.” (P10)

With a common goal and good interaction between lecturers, ‘being-for-oneself’ and ‘being-for-others’ become integrated as a mutual awareness of the needs of all team members. Mutual respect between colleagues also enhances collegiality, ultimately leading to lecturers perceiving that their professional dignity is being upheld:

“Yes, the colleagues, we respect each other… we give each other the opportunity to deliberate until we reach an agreement.” (P1)

“The way I’m being communicated to should be with respect…..even though we may not agree on some things but… to be corrected in, a respectable way makes me feel, my professional dignity is enhanced.” (P3)

“I feel that with colleagues we treat each other well. It’s both ways, you treat people well (you respect them), they treat you well.” (P11)

The participants who respected their colleagues experienced optimal collegiality to the benefit of the preservation of their professional dignity. Their colleagues showed respect for them as lecturers and the contribution that they make to the professional growth of students. Without collegiality in a workplace interpersonal conflict may develop.

## Constituent: creating conducive teaching environments

In nursing education, the working environment needs to be conducive to teaching and learning to the benefit of the students’ growth and development. Such environments may also have a positive effect on the interaction between lecturers and with students. The participants had mixed feelings about the working environment and how it influenced their professional dignity. One participant was satisfied with the working environment:

“I have my own office, open space, the students can come to consult… and the skills lab.… has sufficient resources.” (P16)

Some participants were not satisfied with the working environment:

“It’s frustrating especially with the resources that are not functioning or are not available.” (P4)

“When it comes to resources, I don’t think we are given enough.” (P11)

An empowering working environment and the availability of quality resources may contribute to the enhancement of lecturers perceived professional dignity. On the other hand, may a poorly resourced environment be detrimental to the perceived professional dignity of nursing lecturers.

## Constituent: being respected by students

Students who recently completed secondary school education as well as older students who further their studies are registered at the designated nursing education institution. According to the participants, the older students showed more respect to them than the younger students. Lecturers’ professional dignity can be compromised during encounters with students who are disrespectful towards them. The participants found that their perceived professional dignity was influenced both, positively and negatively by their students:

“With interaction with students, it varies, the younger ones…. I think the soft skills are not very well developed…. You’ve got to remind them about, etiquette and the lines of communication.” (P3)

“Once I address students with dignity, with respect, they also gave me the same.” (P5)

“On an individual basis, students do respect you… Our big classes, like 250 students in class they tend to keep doing whatever they want in class and not listen to you, which for me is disrespect.” (P2)

“This is really difficult because of the big classes… Some students are very respectful, in other cases not so much.” (P8)

Students who respected the participants contributed to their perceived professional dignity.

## Constituent: perceiving lecturers as possessing integrity

The participants believed that they acted with integrity to meet the educational needs of their students. They expected their students to possess integrity too. The same applied to the interaction between the participants and the management of the designated education institution. They hoped that management would trust them, but they often experienced that they had not been trusted to perform their work independently. According to the participants, their professional dignity got jeopardized when management did not perceive them as people with integrity:

“I believe you can’t compromise your dignity… for me, my dignity goes with my integrity.” (P13)

“We have a register where we need to sign in and out every day… so that they can monitor us as if we are not able to account for ourselves.” (P12).

“When I go and submit work to my manager, she’ll say let me confirm with your colleagues whether you did this…whether it is correct before I can acknowledge it.” (P16).

In order for lecturers to perceive their professional dignity in a positive way, they need to be allowed to work independently and to be perceived as dignified professional people. They need to be trusted to do the required work without being supervised and monitored all the time.

## Discussion

Shortages of nursing lecturers often occur to the extent that education institutions have to turn prospective students away.^28^ It contributes to the dire nurse shortages that are globally experienced.^29^ It seems as if people are getting used to nurse shortages and it therefore does not receive significant attention in the media. The healthcare needs of societies, however, puts extreme pressure on education institutions to increase the number of students to ensure that the shortages of nurses could soon be addressed. It happens at a time when nursing education institutions struggle to appoint sufficient lecturers.^28^ The limited number of lecturers therefore experience work overload and often feel under-valued and disrespected. Some of the participants felt that it did not really matter that they were recognised as hardworking and caring professionals who were sacrificing much to support students to grow professionally. They were despondent that managers and students at times disrespected them. The participants felt to instil attitudes for honouring the perceived professional dignity of nursing lecturers, it is essential to acknowledge them as nursing specialists. Nursing lecturers’ professional image as specialists may contribute significantly to how they perceive their professional dignity, and it may correlate positively with their quality of work.^7,8^ Nursing lecturers should feel valued and respected by their managers for attaining specialised certification in education.^30^

The participants wished to be allowed to function autonomously as competent staff of the designated nursing education institution. They wanted to be trusted by management and their professional dignity acknowledged. Self-determined people do not need ongoing supervision.^31^ Self-determination aimed at personal growth and development, focuses on individual psychological needs of autonomy, competence and relatedness as part of motivation.^32^ The human right to self-determination is acknowledged by international law.^33^ Self-determination capabilities of lecturers should be valued and be further developed to empower them to excel as dignified lecturers.^34^ Lecturers’ capabilities refer to their teaching, communication and technical skills as well as their higher cognitive abilities.^35^ The acknowledgement of nursing lecturers’ capabilities may lead to increased self-confidence which is a prerequisite for their enhanced professional dignity.^4,14^ Unfortunately, the opposite may also happen. A lack of trust from management may lead to lecturers’ poor self-esteem and may result in negative perceptions of their professional dignity.^4,14^ The participants wanted to be acknowledged for their capabilities as it made them feel valued and it boosted their professional dignity.

Collegiality within an empowering working environment encompasses supportive and positive relationships between employees.^7^ According to the participants, collegiality played a vital role in their perceived professional dignity. They also wished for better support from management as they believed that it could have led to an empowering working environment. Interaction between colleagues may have negative or positive consequences for the self-esteem and professional dignity of all employees. The same applies to nursing lecturers. Collegial solidarity,^36^ is beneficial to employees’ dignity when it leads to mutual support and the development of close professional bonds. When less stress is experienced, and employees trust their colleagues, job satisfaction and collegial support are common.^37^ The participants valued the close bond among the lecturers in the designated institution. They experienced cohesion with their colleagues due to the shared goal of training competent and caring professional nurses. With good interpersonal relationships and mutual trust, nursing lecturers become more than just collaborative colleagues, they become a team, demonstrating peer support to form a social solidarity that may lead to motivate them to become experts in teaching future professional nurses.^38^

People who feel respected by others experience dignity.^2,8^ Peoples’ dignity can therefore be enhanced through the respect that they receive from others.^39^ The participants of this study were convinced that when their students showed respect to them, their perceived dignity was enhanced. People can learn to interact respectfully with others and to uphold their dignity.^40^ It’s the responsibility of nursing lecturers to show their students how to interact with others in a respectful manner. Respectful interaction between students and lecturers can have a significant impact on how lecturers perceive their professional dignity. When students and lectures expect one another, lecturers’ ‘being-for-others’ and their ‘being-for-oneself’ may converge to form ‘being-for-ourselves’.^27^

This study refers to the subjectivity of perceptions of dignity. It is therefore possible that the factors that influence the perceived dignity of nursing lecturers at the designated nursing education institution may be different from the factors that influence nursing lecturers’ professional dignity at other institutions. The findings of the study are, however, significant as it emphasizes the importance of trusting relationships and positive working environments to enable nursing lecturers to perceive professional dignity. There were no contextual or cultural limitations.

## Conclusion and recommendations

The disclosed essence refers to ‘longing for professional dignity’. Lecturers want to feel dignified and respected as educational specialists. As professional people with integrity they are capable of working autonomously and do not need ongoing supervision by institutional management. The appreciation of their capabilities by management, colleagues and students contribute to their professional dignity. They depend on the collegiality of fellow lecturers to optimally contribute to the professional development of their students. Conducive teaching environments support lecturers’ achievements. Institutional policies to create respectful working environments may contribute to dignifying relationships between management, lecturers and students to cultivate future professional nurses who respect themselves and others.

## Data Availability

The data that support the findings of the study are available from the corresponding author, upon reasonable request.*
